# An immunohistochemical approach to cell wall polysaccharide specialization in maritime pine (*Pinus pinaster*) needles

**DOI:** 10.1007/s00709-025-02041-5

**Published:** 2025-02-18

**Authors:** Santiago Michavila, Antonio Encina, Alfonso G. De la Rubia, María Luz Centeno, Penélope García-Angulo

**Affiliations:** 1 Grupo de Investigación de Fisiología y Biotecnología de Plantas (FISIOVEGEN), Departamento de Ingeniería y Ciencias Agrarias, Facultad de Ciencias Biológicas y Ambientales, Universidad de León, Área de Fisiología Vegetal, Campus de Vegazana s/n, 24071 León, Spain; 2 Genómica y Proteómica (INBIOMIC), Instituto de Biología Molecular, Universidad de León, Campus de Vegazana s/n, 24071 León, Spain

**Keywords:** Maritime pine, *Pinus pinaster*, Needles, Cell wall specialization, Immunohistochemistry

## Abstract

Pine needles are specialized leaves bearing distinctive anatomical features whose function is to minimize water loss. At the molecular level, needle specialization is expected to lead to heterogeneity in cell wall (CW) composition. By immunohistochemical analysis of CW epitopes in maritime pine (*Pinus pinaster*) needles, we described the variability of CW composition in all tissues, discussing its possible relationship with the cell function. Cells specialized in water transport, tracheids, transfusion tracheids, and endodermis, together with epi/hypodermal and stomatal cells, had similar CW composition, where epitopes for pectins (homogalacturonan and rhamnogalacturonan-I), xyloglucan, and heteroxylans were abundant. Heteroxylans differ between the dermis, enriched in arabinoxylan, and endodermis, enriched in glucuronoxylan. Xylem CWs were very rich in (arabino)xylans and homogalacturonan and distinctively lacked immunolabelling for rhamnogalacturonan-I. CWs from phloem, transfusion phloem, mesophyll, and resin ducts were enriched in homogalacturonan, rhamnogalacturonan-I, and xyloglucan, showing a low content for heteroxylan epitopes. Arabinogalactan proteins were detected in mesophyll and in cells specialized in solute transport such as phloem, duct sheath, and guard cells. These results show that tissues with similar functions have similar polysaccharide composition, shedding light on the role of CW components through different tissues.

**Supplementary Information:**

The online version contains supplementary material available at 10.1007/s00709-025-02041-5.

## Introduction

The plant cell wall (CW) is a structure which surrounds the protoplasm of plant cells providing shape and tensile strength, playing key roles in cell-to-cell communication or transport, and also participating in signal transduction during development or in response to external cues (Keegstra 2010). Cell and tissue specialization involves changes at the molecular level in the composition and structure of the CW, which can also occur in plants adapted to different environments and stress situations. This CW reorganization is species- and tissue-specific and is regulated temporarily and spatially within a single cell (Popper et al. 2011; Dauphin et al. 2022). Studying concrete examples of adaptation and how a particular species has coped helps us to better understand the relationship between some CW components and the specialization undergone by different cells to carry out their functions. The primary CWs of gymnosperms are similar to those found in dicotyledons and non-grass monocotyledons, and it is composed of a major load-bearing scaffold of cellulose microfibrils embedded in a matrix of hemicelluloses (such as fucoside-xyloglucan, glucomannans, and heteroxylans) and pectins (such as homogalacturonan [HG], rhamnogalacturonans I [RGI] and II [RGII]) (Popper and Fry 2003; Popper and Tuohy 2010; Fangel et al. 2012). Other important components of the CW are arabinogalactan-proteins (AGPs), a special group of highly glycosylated glycoproteins with signaling functions, which seem to be ubiquitous in land plants (Baumann et al. 2021). In addition, gymnosperms contain a high amount of lignin in the secondary CWs, derived primarily from guaiacyl monomers (Ros Barceló 2005; Weng et al. 2008;), which renders the CWs hydrophobic and highly resistant to physical and chemical attacks, such as those produced during drought or biotic stress (Bonello and Blodgett 2003; Choi et al. 2023).

The specific CW composition and structure have been extensively studied in pine wood, but the number of studies performed on needles with this aim is still limited. An example is the work of Donaldson and Williams (2018), who investigated the presence of suberin, lignin, ferulates, and flavonoids associated with CWs during the development of *Pinus radiata* needles, detecting the autofluorescence of these compounds under confocal microscopy. The research methods commonly used to address the problem have been techniques based on quantification of the main CW components or specific epitopes after fractionation, without discriminating against the different tissues (Pattathil et al. 2017; Sun et al. 2023; Wang et al. 2023), or indirectly through gene expression (Nairn et al. 2008; Zamora-Ballesteros et al. 2021; Webster et al. 2022). The immunohistochemical approaches are also very useful as they allow the identification of CW components in each cell type in muro, without chemical alterations. However, they also have some limitations, such as the diversity of cell types, variations in CW thickness, accessibility of epitopes for the antibodies, and subjectivity of the microscopy techniques among others. In any case, deepening into the exact CW composition within different needle tissues could help us not only to gain basic knowledge but also to better understand the role of each component in the function of each cell.

The aim of this work is to determine the topology of the main CW components in needles of maritime pine (*Pinus pinaster*). This organ is of special interest due to the complex composition of its tissues, which share characteristics of both leaves and roots. Accordingly, a wide array of antibodies has been used against specific epitopes of polysaccharides and proteins of the CW, and the relative abundance and distribution of each epitope were estimated. Despite all the limitations of the immunohistochemical approach, this analysis showed interesting results as it seems to relate the epitope distribution maps of the CW components in the needle to the functional specialization of cells.

## Material and methods

### Plant material

Needles were collected from the basal zone of two 60-year-old maritime pines (*Pinus pinaster* Ait.) located in Posada de la Valduerna (León, Spain) (coordinates: 42°27′85.11″ N, 6°04′69.59″ O). Both pines grew under similar environmental conditions, and needles were taken from the southeast-facing side of the tree (Supplementary Fig. 1).

### Biochemical analysis of cell walls

CW isolation was carried out following Rebaque et al. (2017) with some modifications. A set of 20 needles from each pine was separately dried at 60 °C until the mass remained constant, and then the dry material was powdered using a grinder. The powdered dry material was incubated for 24 h in 70% ethanol in a 1:10 (w:V) proportion in agitation (× 3). The alcohol insoluble residue (AIR), obtained after centrifugation at 3220 g for 5 min, was incubated with 2.5 U/mL α-amylase obtained from porcine pancreas (Sigma type VI-A) in 0.01 M phosphate buffer pH 7.0 for 24 h at 37 °C (× 2). After centrifugation, the remaining pellet was incubated with phenol:acetic acid:water (2:1:1, V:V:V) mixture for 8 h at room temperature (× 2). The final residue obtained after centrifugation was washed with 70% ethanol (× 3) and acetone (× 3) and dried at 37 °C until its mass remained constant.

Cellulose was quantified in crude CWs by the Updegraff method (Updegraff 1969), using the hydrolytic conditions described by Saeman et al. (1963). Free hexoses released were quantified with the anthrone method (Dische 1962). Lignin was quantified following the Klason method according to Dence et al. (1992) with minor modifications (Rebaque et al. 2017). CW-esterified ferulic acid (FA) and *p*-cumaric acid (pCA) were analyzed from CWs—after 2 N NaOH saponification, acidification, and partition with ethyl acetate—by high-performance liquid chromatography (HPLC) based on a method previously described by Martínez-Rubio et al. (2020).

To analyze total sugar content and uronic acids, the CWs were first fractionated according to the protocol described by Rebaque et al. (2017) with some modifications. Crude CWs were extracted (150 mg/15 mL) at room temperature with 50 mM cyclohexane-trans-1,2-diamine-*N*,*N*,*N*′,*N*′-tetraacetic acid sodium salt (CDTA) at pH 6.5 for 8 h. After centrifugation at 1811 g for 5 min, the supernatant was collected, and the pellet was washed and suspended with 15 mL of distilled water. The suspension was again centrifuged, and both supernatants obtained were pooled together, which were considered the CDTA fraction. The pellet obtained after CDTA extraction was treated with 15 mL of 0.1 M KOH + 20 mM NaBH4 for 24 h at room temperature, repeating then the same centrifugation and washing process as the previous fractionation and obtaining the 0.1 M KOH fraction. This process was repeated with the pellet but using 15 mL of 4 M KOH + 20 mM NaBH4 obtaining the 4 M KOH fraction. Both KOH fractions were acidified to pH 5.0 with glacial acetic acid. The pellet obtained was resuspended in 10 mL of distilled water and then centrifuged in the same way, and the supernatant was collected as a supernatant-cellulose residue (SnCR) fraction. The final pellet was dried at 60 °C until stable weight, and it was hydrolyzed with 2 mL of 2 N trifluoroacetic acid (TFA) for 2 h at 121 °C, obtaining the TFA fraction.

The total sugar content in each CW fraction was assayed by the phenol–sulfuric acid method (Dubois et al. 1956), using glucose as the standard. Uronic acids were measured using the Blumenkrantz and Asboe-Hansen (1973) method with glucuronic acid as standard (*y* = 0.0078x + 0.0074). For the calculation of neutral sugars, the content of uronic acids was subtracted from total sugars.

### Microscopy and immunohistochemistry

The immunolabelling assay was based on the García-Angulo et al. (2006) protocol. Needles were fixed in 2.5% (w/V) paraformaldehyde in 0.1 M phosphate buffer pH 7.4 at 4 °C for 4 days. After washing twice with 0.1 M phosphate buffer pH 7.5, tissues were dehydrated in an increasing ethanol series (10, 20, 30, 50, 70, 90, and 96% (w/V)), and then they were kept overnight in ethanol:LR White resin (London Resin, Reading, UK) (1:1, V/V) overnight at 4 °C. After that, samples were placed into gelatin capsules containing the LR White resin and allowed to polymerize at 37 °C for 5 days. Sections (1 µm thick) were obtained by an ultracut microtome LKB 2088 (Reichert-Jung, Vienna, Austria) and applied to multi-well slides (ICN Biomedicals, Cleveland, OH, USA) coated with VecTabond reagent (Vector Laboratories, Burlingame, CA, USA).

For the histological study, preliminary sections were obtained, which were stained with 0.4% (w/V) toluidine blue in 1% (w/V) sodium borate solution. These sections were observed on an Olympus BX61 microscope (Olympus, Tokyo, Japan).

In order to locate CW epitopes, serial sections were incubated for 2 h in phosphate-buffered saline (PBS: 0.14 M NaCl, 2.7 mM KCl, 7.8 mM Na2HPO4; 12 H2O 1.5 mM KH2PO4, pH 7.2) with 4% (w/V) fat-free milk powder (MPBS) containing the primary monoclonal antibodies (Table 1) at a 1:10 dilution. After washing exhaustively with PBS, samples were incubated for 2 h in darkness with a 1:100 dilution of an anti-rat immunoglobulin G linked to fluorescein isothiocyanate (Sigma) in MPBS at room temperature. After that, sections were washed with PBS, and they were stained for 10 min in darkness with 0.005% (w/V) calcofluor white solution (fluorescent brightener 28, Sigma) for detecting cellulose. Finally, sections were again washed with PBS and mounted in a glycerol/PBS-based antifade solution (Citifluor AF1) before they were analyzed and observed on an Olympus BX61 microscope equipped with epifluorescence by using an FT-510-LP-520 (450–490 nm) filter—to observe green emission of fluorescein isothiocyanate—and a DAPI filter (FT-358-LP-461) for detecting calcofluor.

**Table 1:** Antibodies used for plant cell wall immunolabeling, indicating the cell wall component, the epitope that is recognized, and the reference

Cell wall component	Antibody name	Specificity/epitope	Reference
Pectin	JIM5	Anti-homogalacturonan (unesterified, partially esterified)	Clausen et al. 2003
	JIM7	Anti-homogalacturonan (methyl esterified)	Clausen et al. 2003
	LM5	Anti-(1 → 4)-*β*-galactan RGI	Jones et al. 1997
	LM6	Anti-(1 → 5)-*α*-arabinan RGI	Willats et al. 1998
Hemicellulose	LM15	Anti-xyloglucan (XXXG structural motif)	Marcus et al. 2008
	LM10	Anti-(1 → 4)-*β*-xylan	McCartney et al. 2005
	LM11	Anti-(1 → 4)-*β*-xylan/arabinoxylans	McCartney et al. 2005
	LM28	Anti-glucuronoxylan	Cornuault et al. 2015
Protein	LM1	Anti-extensin; HRGP	Smallwood et al. 1995
	LM2	Anti-arabinogalactan protein	Smallwood et al. 1996
Others	LM12	Anti-ferulated polysaccharides	Pedersen et al. 2012

Control sections incubated with secondary antibody but without primary antibody were observed under the same filters (Supplementary Fig. 2). Unspecific labeling of secondary antibodies was not detected in any case. The observed fluorescence was standardized into four levels according to whether there was none (0); whether it was very scattered, heterogeneous, and of low intensity (1); whether it was homogeneous and of medium intensity (2); or whether it was not only homogeneous but also of very high intensity (3). The semi-quantification of the fluorescence was made in at least three sections for each antibody (for a detailed explanation, see Supplementary Fig. 3), and it was used for a clusterization analysis (Supplementary Fig. 4).

### Statistics, image analyses, and heatmap

When indicated, Student’s *t*-test or ANOVA (*p* < 0.05) was used for variance analysis using the R 3.6.3 program (R development Core Team 2020). In all images edited, the non-fluorescence background was removed using the Magic Wand Tool within Adobe Photoshop CC2014. The fluorescence areas (green) were overlapped with the same images taken with the DAPI filter for calcofluor staining (blue); unedited images can be found in the Supplementary material (Supplementary Figs. 5 to 7). The standardized values obtained from immunohistochemistry analysis were processed in a heatmap and clustered with R 3.6.3 (R development Core Team 2020), using library ggplot2 (Wickham 2016).

## Results

### Anatomy and cell wall composition of *P. pinaster* needle

**Fig. 1:**
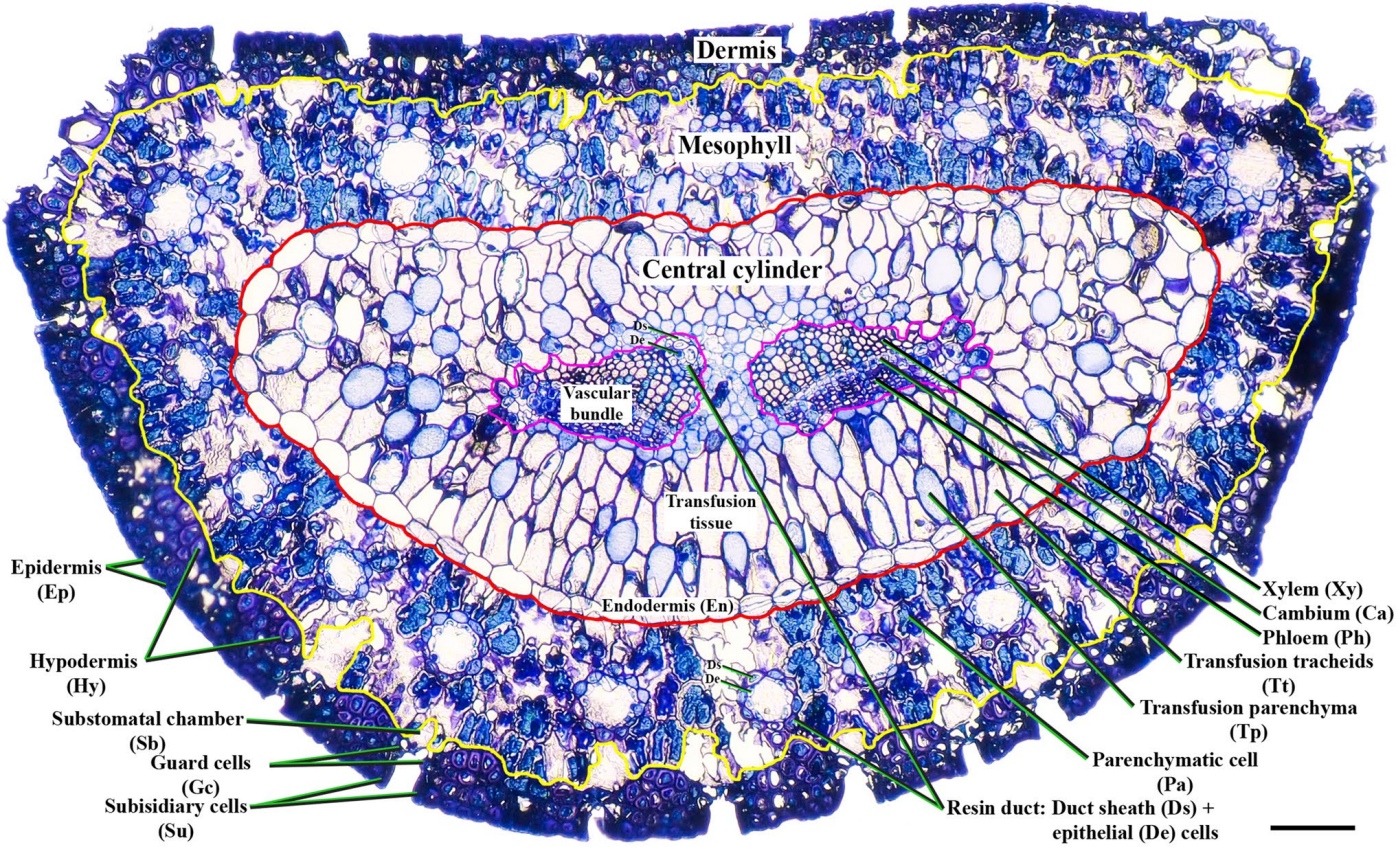
General section of a maritime pine (*Pinus pinaster*) needle stained with blue toluidine, indicating all tissues studied in this article, from outside to inside: the dermis with epidermis (Ep), hypodermis (Hy), and stoma formed by subsidiary (Su) and guard cells (Gc) with substomatal chamber (Sb) below; the mesophyll (between the yellow and red lines) with parenchymatic cells (Pa) and resin ducts; the central cylinder (surrounded in red) consisting of the endodermis (En), vascular bundle—xylem (Xy), cambium (Ca) and phloem (Ph), small resin ducts—sheath (Ds), and epithelial cells (De), surrounded by the transfusion tissue—transfusion tracheid (Tt) and transfusion parenchyma (Tp). Scale bar 100 µm

Placing the adaxial side on top, the tissue distribution of a pine needle from outside to inside is as follows: the dermis (outside layer of the yellow line), the mesophyll (intermediate layer, between yellow and red lines), and the central cylinder (inside the red line) (Fig. 1). Inside the dermis, both an epi- (Ep) and a sclerenchymatous hypodermis (Hy) can be distinguished. This dermis is usually covered by a thick wax cuticle. Sunken stomata are evenly distributed across the dermis. Stomatal apparatus is formed of subsidiary (Su), which includes both lateral epidermal and polar epidermal subsidiary cells, and guard cells (Gc) communicated with a substomatal chamber (Sb) (Fig. 1). Below the dermis a thick mesophyll tissue is located surrounding the central cylinder. The mesophyll is formed of homogeneous parenchymatic cells (Pa) characterized by their infolded shape (armed mesophyll cells). Dispersed in the mesophyll, numerous resin ducts can be distinguished. Resin ducts are lined by a monolayer of epithelial (De) cells encircled in duct sheath (Ds) cells (Fig. 1). The central cylinder consists of two collateral vascular bundles formed of xylem (Xy), cambium (Ca), phloem (Ph), and small resin ducts like those found in the mesophyll with both sheath (Ds) and epithelial cells (De). Vascular bundles are surrounded by the transfusion tissue formed by transfusion tracheids (Tt) and transfusion parenchyma (Tp). The central cylinder is encircled by a continuous layer of endodermal cells (endodermis, En) (Fig. 1).

Compositional analysis of CW extracted from pine needles (Supplementary Fig. 8) showed that almost half of the crude CW was composed of cellulose (47%), followed by lignin (35%) and matrix polysaccharides (20%). CW-esterified phenolics accounted for 3.5% of pine needle CW. Coumaric acid was 10 times more concentrated than ferulic acid. CWs purified from pine needles were fractionated into pectins extracted with CDTA; loosely and tightly bound hemicelluloses were extracted with 0.1 and 4 M KOH, respectively, and polysaccharides tightly bound to cellulose were collected in the Sn-CR fraction. After this CW fractionation, most neutral sugars were obtained in 0.1 M KOH fraction, followed by 4 M KOH and CDTA fractions with a similar content. As expected, most uronic acids were extracted in the pectin-rich CDTA fraction, being 2.1 and 4 times more abundant than in hemicellulosic fractions (0.1 M KOH and 4 M KOH, respectively). The Sn-CR fraction accounted for a low amount of matrix polysaccharides.

### Mapping the cell wall components across *P. pinaster* needles by immunolocalization

#### Mapping the pectins

**Fig. 2:**
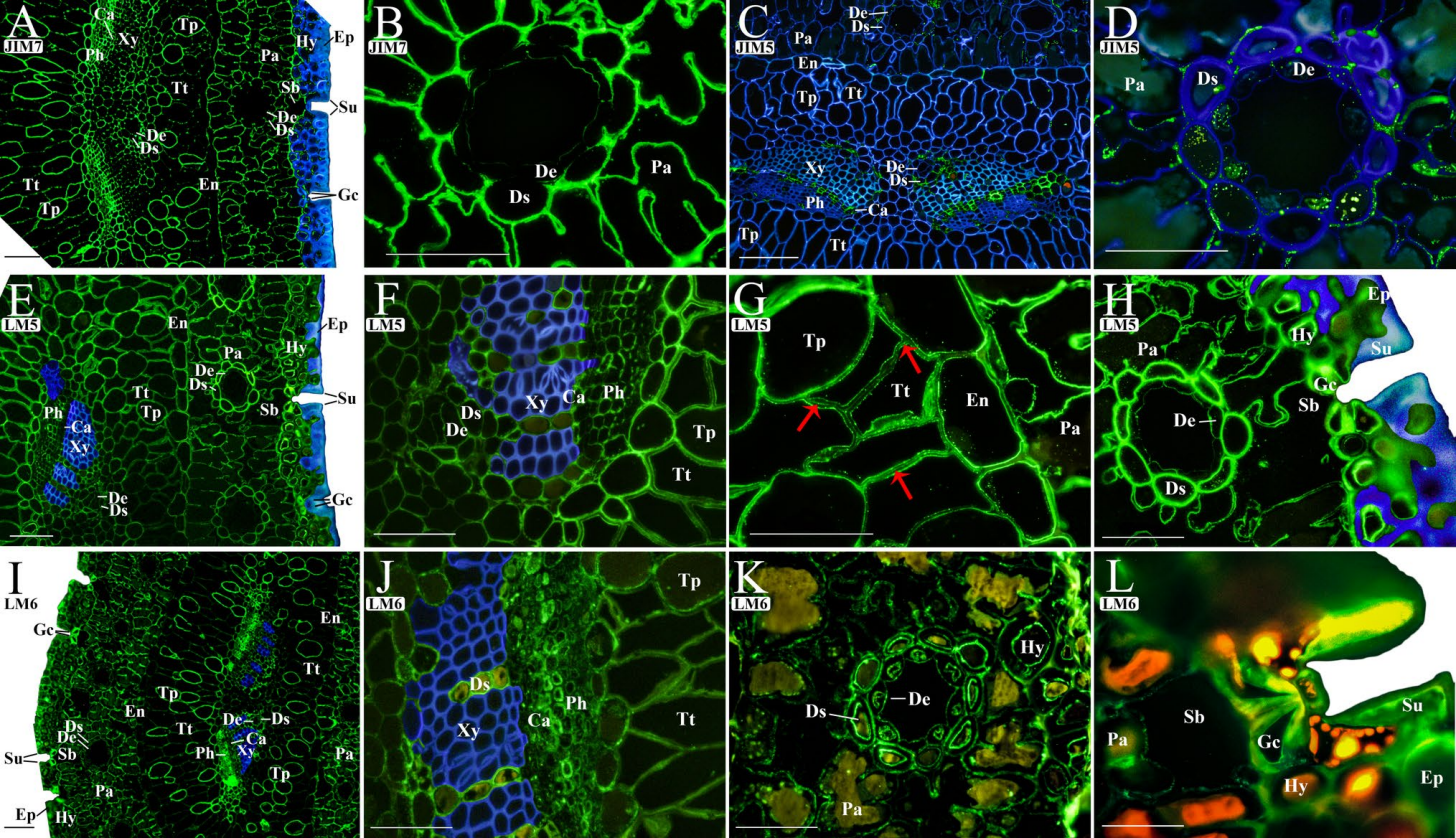
Immunolocalization of pectin polysaccharides in transverse sections of maritime pine (*Pinus pinaster*) needles using antibodies that specifically labeled homogalacturonan with high (JIM7 **A** general section, **B** mesophyll) and low (JIM5 **C** general section, **D** mesophyll) degree of methyl esterification, and (1 → 4)-β-galactan (LM5 **E** general section, **F** vascular bundle, **G** endodermis, **H** mesophyll and dermis) and (1 → 5)-α-arabinan (LM6 **I** general section, **J** vascular bundle, **K** mesophyll, **L** dermis) side chains of rhamnogalacturonan I. The fluorescence due to the antibody is shown in green, and the *β*-glucans stained with calcofluor are shown in blue. Images (**A**, **C**, **D**, **E**, **F**, **H**, **I**, and **J**) have been edited as described in the “Material and methods” section using the same image taken with the calcofluor filter (blue) to better visualize the binding antibody (green) (the unedited images can be found in Supplementary Fig. 5). The red arrows indicate immunolabeling in the middle lamella. Abbreviations: (Ca) cambium, (De) duct epithelial cell, (Ds) duct sheath cell, (En) endodermis, (Ep) epidermis, (Hy) hypodermis, (Gc) guard cell, (Pa) parenchymatic cell, (Ph) phloem, (Sb) substomatal chamber, (Su) subsidiary cell, (Tp) transfusion parenchyma, (Tt) transfusion tracheids, and (Xy) xylem. Scale bars of **A**, **C**, **E**, and **I**, 100 µm, and for **B**, **D**, **F**, **G**, **H**, **J**, **K**, and **L**, 50 µm

After studying the composition of the needle CW, an immunohistology analysis was carried out to determine the epitope distribution in each tissue. Results obtained for the epitopes related to each CW component are described in the following subsections, starting with pectins. The anti-methyl-esterified HG detected by the JIM7 antibody was observed in CWs of all tissues. Nevertheless, the fluorescence intensity in CWs of the hypodermis was rather low (Fig. 2A), and subsidiary cells did not show JIM7 labeling (Su, Fig. 2A) in their CWs. On the contrary, all mesophyll cells presented a high JIM7 immunolabeling in CWs, except the epithelial cells of the ducts (De) (Fig. 2B). Moreover, CWs of the central cylinder tissue presented apparent JIM7-bonding, being the fluorescence more intense in CWs of xylem (Xy), cambium (Ca), phloem (Ph), and transfusion parenchyma (Tp), whereas it was less intense in CWs of transfusion tracheids (Tt) and endodermis (En).

The anti-low methyl-esterified HG recognized by the JIM5 antibody was not found in CWs of dermis cells. In the mesophyll, CWs of parenchymatic cells (Pa) were weak and irregularly labeled with JIM5 (Fig. 2C). Moreover, the JIM5-labeling in CWs of resin ducts was restricted to epithelial cells (De) and to intracellular bodies of sheath cells (Ds) (Fig. 2C, D). JIM5 immunolabeling was observed preferentially in CWs of the central cylinder, especially in the cambium (Ca) cells, and to a lesser extent, in the duct sheath (Ds), xylem (Xy), and phloem (Ph) cells (Fig. 2C). The (1–4)-β-d-galactan side chains of RGI detected by LM5 were extensively present in CWs of all tissues throughout the needle with high labeling intensity (Fig. 2E–H), except in the epidermis, which showed very weak labeling, and in subsidiary cells (Su; Fig. 2H) and xylem (Xy; Fig. 2E, F), where there was no LM5 binding. LM5 showed the highest intensity of labeling in CWs of the guard (Gc), hypodermal (Hy), and duct (Ds) cells (Fig. 2E, H). In the parenchyma cells of both the central cylinder (Tp) and mesophyll (Pa), in addition to the endodermis (En), the binding was very intense in the inner part of the CW close to the membrane (Fig. 2G). However, in transfusion tracheid (Tt) cells, apart from the binding in the inner part of the CW, a delimited line of binding in cell junctions was also detected (see arrows in Fig. 2G). Anti-(1–5)-*α*-l-arabinan antibody LM6 bound to CWs of all the cells except xylem (Xy; Fig. 2I, J). In the central cylinder, the fluorescence intensity was very high in the CWs of phloem (Ph), transfusion parenchyma (Tp), and resin ducts (Fig. 2J), but it was slightly less intense in the transfusion tracheids (Tt), and even lower in the endodermis (En; Fig. 2I). LM6 was detected in the CWs of duct epithelial and sheath cells (De and Ds; Fig. 2K) as well as in some hypodermis cells with thick CWs (Hy; Fig. 2K). LM6 labeling was also observed in CWs of epidermis (Ep) and stomata, being mainly present in the CW of guard and subsidiary cells (Gc and Sc; Fig. 2L).

#### Mapping the hemicelluloses

**Fig. 3:**
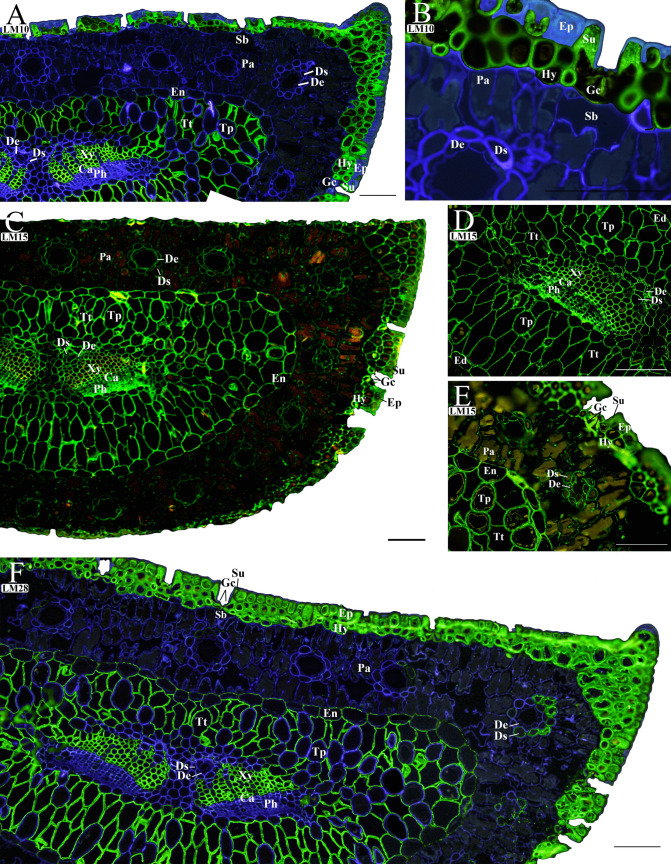
Immunolocalization of hemicellulose polysaccharides in transverse sections of maritime pine (*Pinus pinaster*) needles using the antibodies that specifically labeled xylans (LM10 **A** general section, **B** mesophyll and dermis), xyloglucan (LM15 **C** general section, **D** central cylinder, **E** central cylinder, mesophyll, and dermis), and glucuronoxylan (LM28 **F** general section). The fluorescence due to the antibody is shown in green and the *β*-glucans stained with calcofluor are shown in blue. Images (**A**, **B**, and *F*) have been edited as described in the “Material and Methods” section using the same image taken with the calcofluor filter (blue) to better visualize the binding antibody (green) (the unedited images can be found in Supplementary Fig. 6). The autofluorescence shown in yellow and red is due to lignin, suberin, or another cell wall compound. Abbreviations: (Ca) cambium, (De) duct epithelial cell, (Ds) duct sheath cell, (En) endodermis, (Ep) epidermis, (Hy) hypodermis, (Gc) guard cell, (Pa) parenchymatic cell, (Ph) phloem, (Sb) substomatal chamber, (Su) subsidiary cell, (Tp) transfusion parenchyma, (Tt) transfusion tracheids, and (Xy) xylem. All scale bars 100 µm

The presence of unsubstituted (LM10) and low-substituted (LM11) xylans was analyzed separately, but in this study, both antibodies (LM10 and LM11) showed the same binding patterns. For this reason, we only show LM10 images (Fig. 3A, B) as representative of both antibodies (see LM11 in Supplementary Fig. 9). The LM10/LM11 labeling was very specific to the CWs of hypodermal (Hy; Fig. 3B) and subsidiary cells (Su) in the dermis (Fig. 3A, B) and to the transfusion tracheids (Tt) and the xylem (Xy) cells in the central cylinder (Fig. 3A). LM10-bound epitopes were scarce and unevenly distributed in the CWs of epidermic cells (Ep; Fig. 3B). A total absence of LM10/LM11 labeling was noticed in the CWs of mesophyll cells (Pa) and resin ducts (De, Ds; Fig. 3A, B) and in the endodermis (En; Fig. 3A), cambium, phloem (Ph), and transfusion parenchyma (Tp; Fig. 3A) in the central cylinder. LM15 monoclonal antibody was used to immunolocate xyloglucans (Fig. 3C–E). LM15 epitopes were mainly located in the CWs of the dermis (both Ep and Hy cells) and in the CWs of all cell types in the central cylinder, including endodermis (En) and resin duct cells (De and Ds). LM15 immunolabeling of mesophyll-parenchyma CWs was apparently lower (Pa; Fig. 3C, E). The immunolocation of glucuronoxylan (LM28) showed a similar binding pattern to that found for LM10/LM11. In this case, LM28 labeled the CWs of all the dermis (Ep and Hy), transfusion tracheids (Tt), xylem (Xy), and the endodermic cells (En) of the central cylinder, but neither the mesophyll, the transfusion parenchyma, nor the phloem (Fig. 3F). In addition, the duct sheath (Ds) cells of the mesophyll showed fluorescence, only in those cells with thicker CWs (Fig. 3F).

#### Mapping the glycoproteins and others

**Fig. 4:**
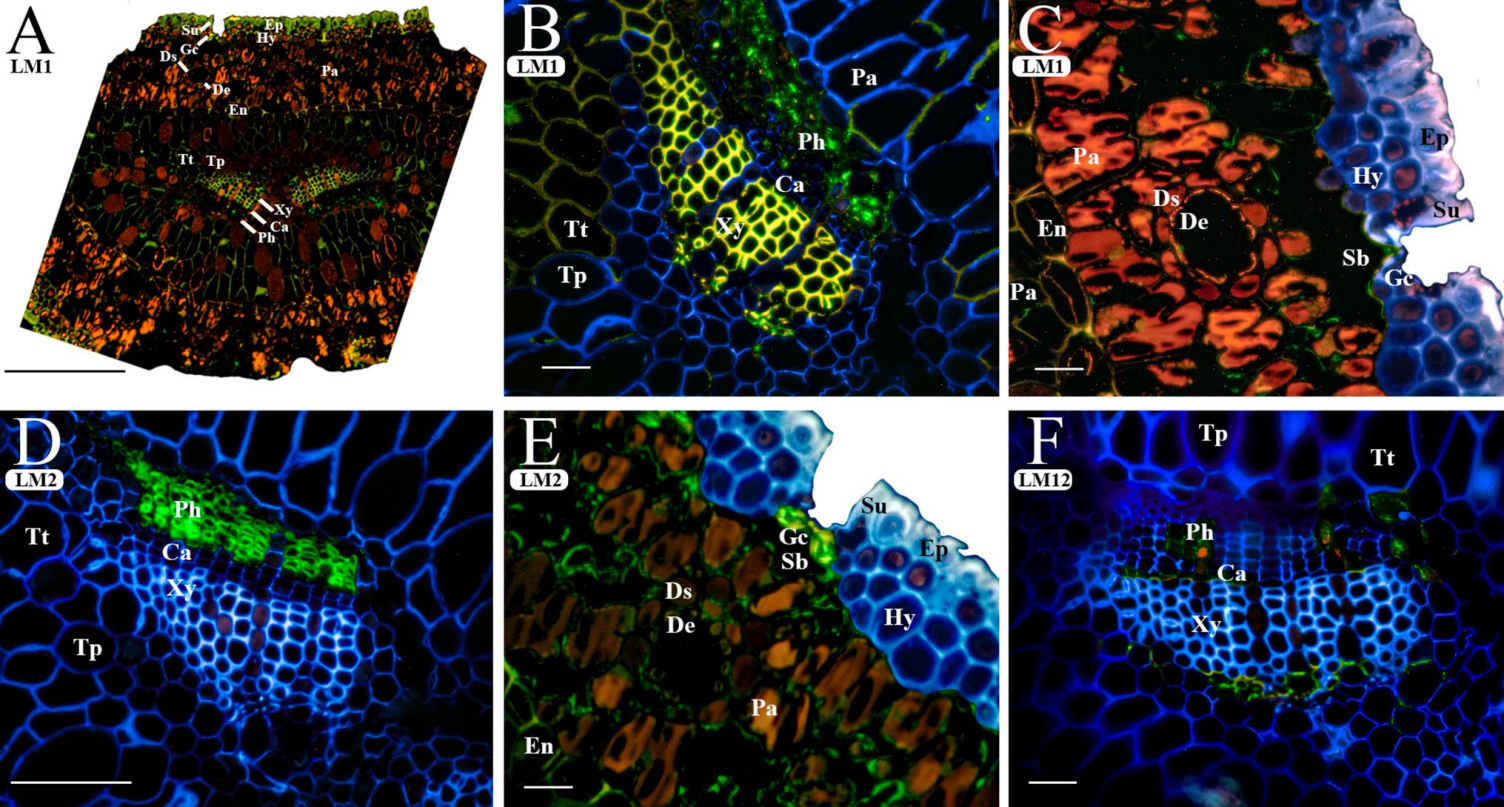
Immunolocalization of cell wall proteins and ferulated polysaccharides in transverse sections of maritime pine (*Pinus pinaster*) needles using the antibodies that specifically label extension (LM1 **A** general section, **B** vascular bundle, **C** mesophyll and dermis), arabinogalactan proteins (LM2 **D** vascular bundle, **E** mesophyll and dermis), and feruloylated polysaccharides (LM12 **F** vascular bundle). The fluorescence due to the antibody is shown in green and the *β*-glucans stained with calcofluor are shown in blue. Images (**B**–**F**) have been edited as described in the “Material and Methods” section using the same image taken with the calcofluor filter (blue) to better visualize the binding antibody (green) (the unedited images can be found in Supplementary Fig. 7). The autofluorescence shown in yellow and red is due to lignin, suberin, or another cell wall compound. Abbreviations: (Ca) cambium, (De) duct epithelial cell, (Ds) duct sheath cell, (En) endodermis, (Ep) epidermis, (Hy) hypodermis, (Pa) parenchymatic cell, (Ph) phloem, (Sb) substomatal chamber, (Su) subsidiary cell, (Tp) transfusion parenchyma, (Tt) transfusion tracheids, and (Xy) xylem. Scale bars of **A** and **D** 100 µm; **D**, **C**, **E**, and **F** 10 µm

Due to the low labeling observed for LM1, LM2, and LM12 antibodies, and the high autofluorescence of both CWs (yellow) and the cytoplasmic content (red or brown) of many cells, we decided to perform a merge of the images obtained for each of these antibodies together with calcofluor (Fig. 4 B–F). In this way, the cells without immunolabelling were stained blue (calcofluor), while those that present immunolabeling were observed in green (FTIC of the secondary antibody), which facilitates their detection. Anti-extensin antibody (LM1) showed scarce binding throughout the CWs of needle tissues, which is why elevated autofluorescence is observed in both CWs (yellow) and cytoplasms (red) of most cells. Extensin epitopes were immunolocated in the CWs (green) of guard cells (Gc), transfusion tracheids (Tt), and, remarkably, vascular bundles associated with both xylem and phloem (Xy and Ph; Fig. 4A–C). Slight labeling was observed in some parenchyma cells, which was masked by the high autofluorescence (detected in red) in the cytoplasm of these cells (Pa; Fig. 4A, C). The fluoresce due to the anti-AGP LM2 was especially intense in the phloem CWs (Ph; Fig. 4D). In addition, LM2 epitopes were immunolocated in the CW of guard cells (Gc; Fig. 4E), and in the sheath cells of the ducts (Ds) and some parenchyma cells (Pa; Fig. 4E) although with very low intensity. Finally, the fluorescence obtained with anti-feruloylated polymers antibody LM12 was observed sporadically in some cambium cells (Ca) and in transfusion tracheids (Tt) of the central cylinder (Fig. 4F).

### Clusterization analysis by heatmap

A heatmap is a bi-dimensional representation of data based on color categories, in which the information is summarized into an interpretative visual graph. To make this categorical classification, an estimation of relative intensity and homogeneity of labeling in each tissue was established with a semi-quantitative scale using four levels (see Supplementary Fig. 3) to detect similar CW-epitope patterns among tissues. The heatmap showed two kinds of clustering: one related to antibodies (A and B), and the other to cell type (C and D) (Supplementary Fig. 4). All antibodies with the lowest labeling were grouped in cluster A, while those antibodies with high labeling intensity were grouped in cluster B. Cluster A1 grouped epitopes of feruloylated polymers, HG with a low degree of methyl esterification, extensins, and AGPs. Cluster A2 grouped xylan/arabinoxylan and glucuronoxylan epitopes. On the other hand, the most abundant epitopes were those related to side chains of RGI, HG with a high degree of methylation, and non-fucosylated xyloglucan (cluster B). According to the tissue-epitope pattern, two clearly differentiated groups were obtained (C and D). Cluster C included all cell types from mesophyll and central cylinder, except transfusion tracheids (Tt), endodermis (En), and xylem (Xy), which were in cluster D together with cell types from the dermis. Inside group C1, duct epithelia (De) and sheath (Ds) cells grouped together in close relation with transfusion parenchyma (Tp) cells. Cambium (Ca) was also located in group C1 but separated from the rest of the cell types. In C2, duct sheath cells (Ds) from the mesophyll were grouped apart from parenchyma (Pa) and phloem (Ph) cells. Inside group D, transfusion tracheids (Tt), hypodermis (Hy), guard cells (Gc), and endodermis (En) were grouped in D1. Epidermis (Ep) and subsidiary (Su) cells were located in cluster D2, and the xylem was separated from the rest in cluster D3.

## Discussion

### Epidermal cells are rich in pectins and hemicelluloses

Epidermal cells were rich in pectic epitopes (Fig. 2), especially in those from side chains of RGI (LM5 and LM6) and methyl-esterified HG (JIM7). This fact is indicative of the presence of a thick primary CW deposited before lignification (Meents et al. 2018). Sclerenchymatous hypodermal cells showed intense labeling for hemicelluloses, which could be related to lignified CWs as expected for cells providing mechanical support to the needle (Meents et al. 2018). In this sense, a strong correlation between lignin and hemicelluloses has been suggested in both hard- and softwood species (Donalson and Knox 2012; Huang et al. 2016). Xylan can constitute up to 15% of gymnosperm (softwood) CWs, and their substitutions could affect the association properties with other polymers such as cellulose or lignin, and hence, the mechanical strength, flexibility, and recalcitrance to enzymatic digestion of the CWs (Terrett and Dupree 2019). The lignification of hypodermal cells could also help to prevent pathogen entry through the CW (Houston et al. 2016).

### Guard cells show a specialized cell wall composition among all epidermal cells

According to our results, epidermal and subsidiary cells presented very similar immunolabeling patterns, except for methyl-esterified HG (JIM7) and galactan side chains of RGI (LM5), whose labeling was not present in subsidiary cells but was present in epidermal cells. Especially, guard cells presented high labeling for LM5- and LM6-RGI epitopes (Fig. 2 and Supplementary Fig. 4). Pectins have been associated with changes in the elasticity, flexibility, or stiffness of guard CWs in different species (Amsbury et al. 2016; Shtein et al. 2017; Jaafar et al. 2024). In fact, they are known to be essential for guard cell movement (Jones et al. 2003, 2005; Chen et al. 2021). Although we cannot conclude further regarding the degree of pectin methyl esterification in needle guard cells, a differential distribution of pectins with high and low degree of methyl esterification in *Arabidopsis* (Jaafar et al. 2024) and in maize guard cells (Gkolemis et al. 2023) has been described, which seems to have mechanical implications. Little is known about the properties or functions of hemicellulose in stomatal complexes (Jaafar et al. 2024) but, according to our results, hemicellulose-immunolabeling patterns were similar to those found in epidermal cells (Fig. 3). However, a distinctive feature of needle guard cells, which undergo rapid changes of turgor pressure, is the presence of AGPs (recognized by LM2) and extensin (LM1) epitopes (Fig. 4A and B), which have been proposed to participate in elongation and extension changes in the CW (Anderson and Kieber 2020). The specific presence of AGPs in guard cells has also been observed during the development of stomatal complexes in maize (Giannoutsou et al. 2016), which could be indicative of a cell specialization marker.

### The abundance of hemicellulose epitopes characterizes the cell wall of water-conducting cells in needles

Xylem cells, transfusion tracheids, and endodermis cells are rich in xylan-related epitopes (LM28, LM10, and LM11) (Fig. 3; Supplementary Fig. 4). The greater presence of hemicelluloses in these cell types than in others of the central cylinder may indicate a higher degree of CW lignification/suberification, even in the case of endodermal cells which differentiate a Casparian strip as demonstrated in *Pinus bungeana* needles (Wu et al. 2003). The endodermis of needles surrounds the central cylinder and is one of the most distinctive features of conifer leaves. As shown in *P. bungeana*, the Casparian strip in needles is more apoplastic-permeable (Wu et al. 2005). The presence of suberin in this context is still a matter of debate (Wu et al. 2003). Recently, it has been reported that glucuronic acid substitutions of xylans are necessary for binding suberin-like compounds to the cell walls on wood (Derba-Maceluch et al. 2023). Glucuronoxylan epitopes were found in endodermis cells (Fig. 3F), suggesting the presence of suberin-like compounds in the endodermis of coniferous needles.

### The richness in pectin and the absence of heteroxylan epitopes characterize the cell types involved in solute synthesis and transport

Mesophyll and central cylinder cell types had CWs richer in pectin epitopes than those in the dermis (Fig. 2; Supplementary Fig. 4). Particularly, CWs from tissues specialized in solute synthesis and transport such as parenchyma cells, phloem, and resin duct cells had the highest immunolabeling for HG (JIM7) and RGI (LM5 and LM6). The epitope distribution pattern found in CWs of phloem and mesophyll parenchyma cells was very similar to those found in other gymnosperm species such as Norway spruce and Scots pine, in which pectin (RGI and HG) and xyloglucan epitopes are abundant. However, there is no presence of heteroxylan epitopes in this species (Kim and Daniel 2017), which indicates a low level of lignification (Donalson and Knox 2012). As it is known, pectins confer strength and elasticity properties (Peaucelle et al. 2011), which, in this case, facilitate CW resistance to high internal pressures associated with loading and mass flow capacity of specialized living cells such as phloem cells (McCubbin and Braun 2021).

Embedded in the mesophyll, numerous resin ducts were observed (Fig. 1). Both mesophyll and central cylinder ducts are composed of the sheath and epithelial cells that have a CW composition similar to that of parenchyma cells, except for the lack of extensin (LM1 labeling) in duct cells*.* The epithelial cells of the ducts from the mesophyll and central cylinder had similar CWs, but the LM6 immunolabeling shown in duct epithelial cells of *P. pinaster* needles could be due to the presence of AGPs in these cells (Pérez-Pérez et al. 2019). AGP proteins are anchored to the plasma membrane, showing 90% of polysaccharides in their composition (Showalter 2001), which is the part of the molecule recognized by the antibodies. Since duct cells appear to be plasmolyzed and LM6 is associated with the plasma membrane (Fig. 2K), this could indicate that the labeling is due to AGPs rather than RGI, in this case.

### Cambium cells are rich in unesterified pectin epitopes

CWs of cambium cells in the central cylinder showed the most intense labeling for unesterified pectin epitopes (JIM5) among all cells of the needle (Fig. 2), which may be correlated with their meristematic activity (Baïer et al. 1994; Somssich et al. 2016). Changes in pectin epitopes have been previously described during vascular cambium development (Liu et al. 2017). Dividing cells are usually rich in pectin with a high level of methyl esterification (Dolan et al. 1997). However, using AFM, McCubbin et al. (2021) found that pectin demethyl esterification was correlated to greater tissue elasticity, a key step preceding the differentiation and organogenesis which take place in the meristem (Peaucelle et al. 2011). In this sense, cambium cells are implicated in vascular tissue (phloem and xylem) formation and for that, cambium cells have a fast transdifferentiation process after division, which could explain the presence of a high amount of unesterified pectins in these cells.

### Transfusion cells in the central cylinder show a distinct immunoprofile depending on their function

Transfusion tracheids and transfusion parenchyma are specialized in the transport of water and solutes, respectively, between mesophyll cells and vascular bundles (Canny 1993). As expected, the epitope distribution of the former is more similar to that observed in the xylem, whereas the polysaccharide composition of the latter is more similar to that found in CWs of phloem cells. This again indicates that the topology clustering of the CW components is related to the function of the cells rather than cell location, which is common to the four cell types.

## Conclusion

In summary, here we have shown the diversity in the CW composition of the highly specialized leaf of *P. pinaster*. To our knowledge, this is the first work providing a map of CW component distribution in such a complex organ. Differential organization of CW components in various cell types and tissues in pine needles suggests specific functional adaptations, shedding light on the role of CW components throughout different tissues. Until individual cell quantification techniques are developed, the results obtained through CW immunohistochemistry have allowed us to carry out this needle characterization. Despite all the limitations of this type of approach, the results obtained illustrate the methodological solutions offered by CW immunocytochemistry, which we believe provides significant insights to the scientific community.

## Supplementary Information

Below is the link to the electronic supplementary material.

Supplementary file1 (DOCX 8.30 MB)
