# Supplementary material for: An immunohistochemical approach to cell wall polysaccharide specialization in maritime pine (Pinus pinaster) needles
Source: Protoplasma. 2025 Feb 18;262(4):979–91. doi: 10.1007/s00709-025-02041-5 (PMC12141410; doi:10.1007/s00709-025-02041-5)
Supplement: Supplementary file 1 — Supplementary file1 (DOCX 8.30 MB) [file 709_2025_2041_MOESM1_ESM.docx]

An immunohistochemical approach to cell wall polysaccharide specialization in maritime pine (*Pinus pinaster*) needles

# Santiago Michavila^1^, Antonio Encina^1,2^, Alfonso G. De la Rubia^1^, María Luz Centeno^1,2^, Penélope García-Angulo^1,2*^

^1^Grupo de investigación de Fisiología y Biotecnología de Plantas (FISIOVEGEN), Departamento de

Ingeniería y Ciencias Agrarias, Universidad de León, Campus de Vegazana s/n, Facultad de Ciencias

Biológicas y Ambientales, Área de Fisiología Vegetal, 24071 León, España.

^2^Instituto de Biología Molecular, Genómica y Proteómica (INBIOMIC), Universidad de León, Campus de Vegazana s/n, 24071, León, España.

Corresponding Author: [penelope.garcia@unileon.es](mailto:penelope.garcia@unileon.es)

**Supplementary Figures**

**A)**


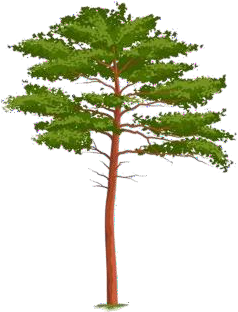

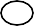

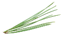

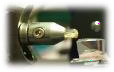

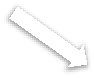

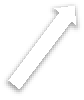

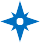


# Cell Wall Extraction


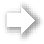

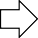

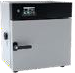

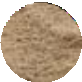

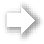

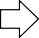

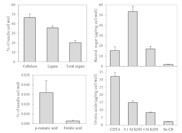


Pool of 20 needles per pine

SE

**B)**

# Microscopy preparation

**C)**


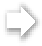

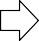


# Cell Wall Composition

**Microscopy and Immunohistochemistry**

x2 pines

42º 27' 85.11" N

6º 04' 69.59" O

At least 3 sections


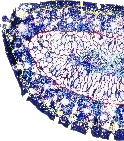

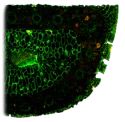


**Supplementary Figure 1. Experimental design for needle analysis** A) Needles were collected from the basal zone of two 60-year-old maritime pines (*Pinus pinaster* Ait.) located in Posada de la Valduerna (León, Spain). Both pines grew under similar environmental conditions and needles were taken from the southeast facing side of the tree. B) For cell wall analysis, a set of 20 needles from each pine was dried, ground and used to analyze cell wall composition. C) The location of the different cell wall components was analyzed by microscopy and immunohistochemistry techniques. For this purpose, at least 3 sections of needles from both pines were analyzed.


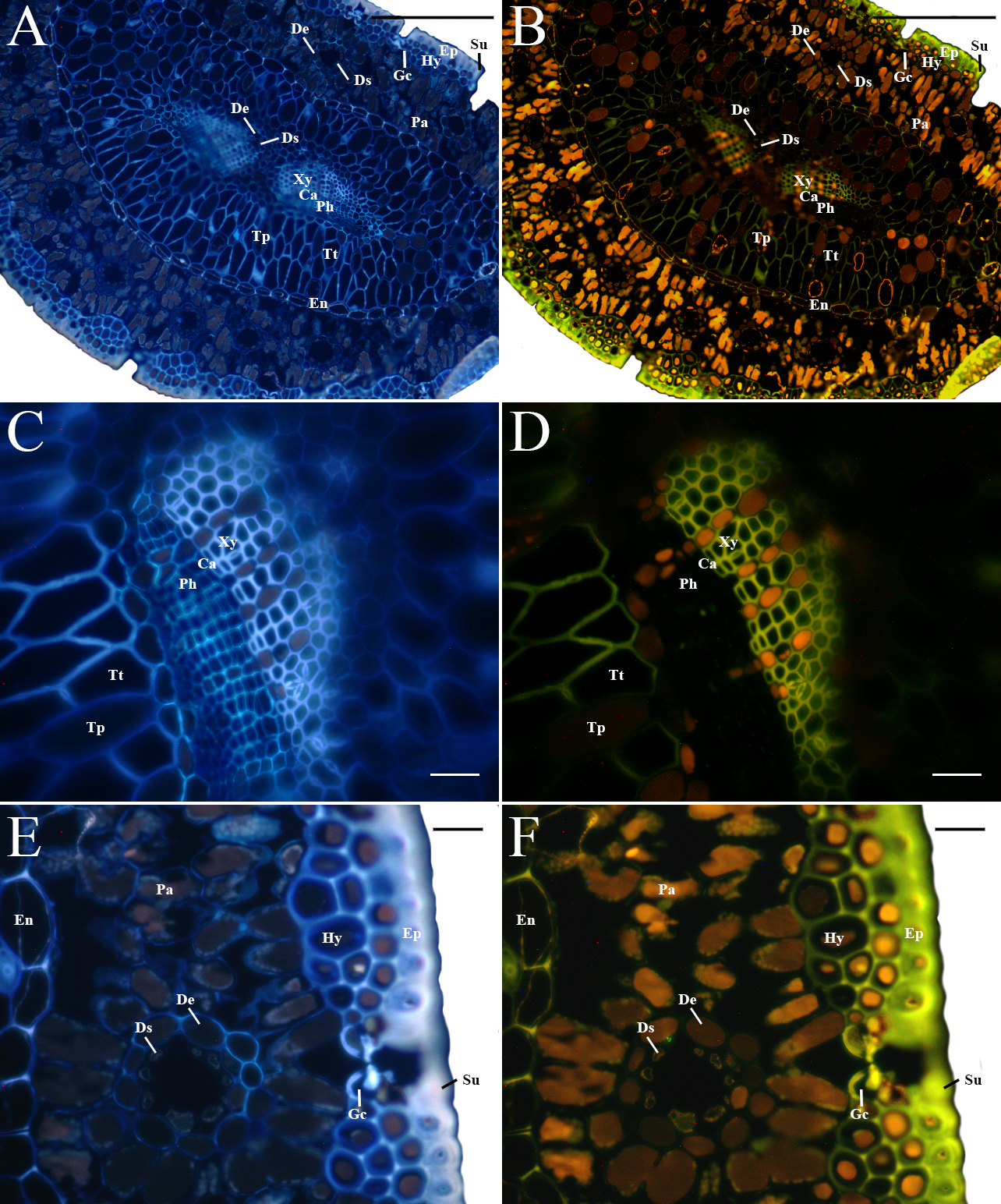


**Supplementary Figure 2.- Control sections of maritime pine (*P. pinaster*) needle** incubated with secondary antibody without primary antibody. Images were taken after Calcofluor staining and observed with DAPI filter (left) or with FTIC filter (right). The autofluorescence due to lignin and another cell compounds are shown in yellow, brown and red colors. Different tissues are shown: (A, B) General section of the needle (see also Fig. 1); (C, D) Section of the central cylinder focused on vascular bundles; (E, F) Mesophyll and dermis of needle. Abbreviations: (Ca) cambium, (De) duct epithelial cell, (Ds) duct sheath cell, (En) endodermis, (Ep) epidermis, (Hy) hypodermis, (Gc) guard cell, (Pa) parenchymatic cell, (Ph) phloem, (Su) subsidiary cell, (Tp) transfusion parenchyma, (Tt) transfusion tracheid and, (Xy) xylem. (A and B zoom 10x; C, D, E and F zoom 40x) (A and B scale bar 100 µM; C, D, E and F scales bars 10 µM).

**
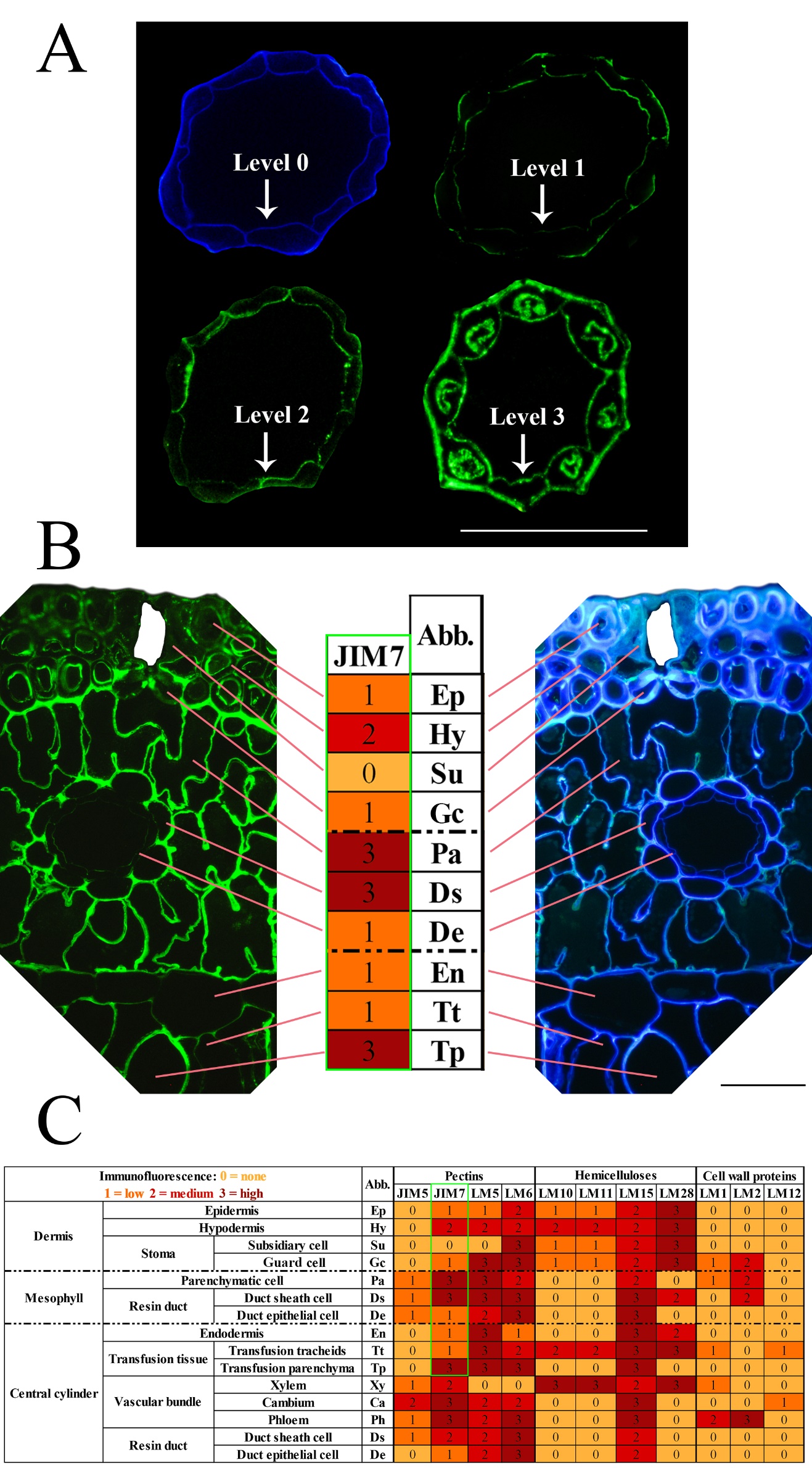
**

**Supplementary Figure 3.- Standardization of fluorescence** by semi-quantification using four levels according to intensity (A) and distribution (B). Example of the **scale relative intensity** (A) with different antibodies (JIM7, LM5 and LM6) in the inner side of the parenchymatic resin duct as an example, where **Level 0** was no fluorescence (calcofluor is shown in blue), **Level 1** was low intensity (JIM7 antibody); **Level 2** was medium intensity (LM5 antibody); and **Level 3** was high intensity (LM6 antibody). (A). Example of **scale distribution** (B) used in different tissues immunolabeled with JIM7 (left) or stained with calcofluor (right) with the assigned level and the abbreviations (middle), where **Level 0** was no fluorescence (see Su cells), **Level 1** was very scattered, heterogeneous and low intensity (see Ep, Oc, De, En and Tt cells); **Level 2** was homogeneous and medium intensity (see Hy cells); and **Level 3** was homogeneous and with high intensity (see Pa, Ds and Tp cells). Scale bar 50 µm. C) **Annotation table** with the mean value of scale estimated for each tissue and antibody of at least 3 sections. These semiquantitative values were used for Heatmap clusterization. The column highlighted in green is the one shown in A.


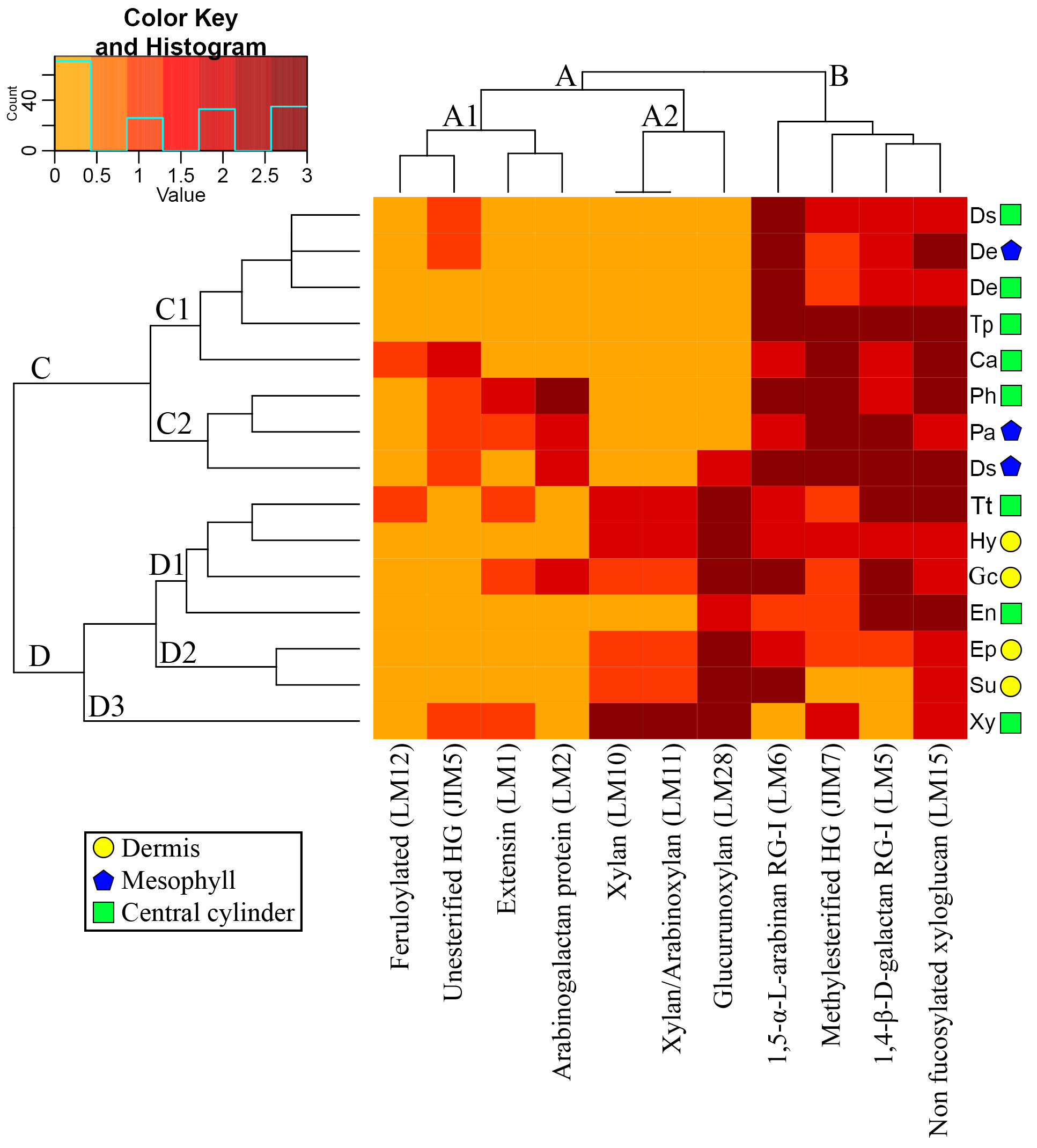


**Supplementary Figure 4.- Heatmap of data obtained from the semi-quantification of *Pinus pinaster* needles immunofluorescence intensity/distribution for each tissue and antibody (see Sup. Fig 3)**. Cell types of each tissue are clustered in horizontal axis, while monoclonal antibodies are clustered in vertical axis. Those cell types belonging to dermis are marked with a yellow circle, mesophyll with a blue pentagon, and central cylinder with a green square. Abbreviations: (Ca) cambium, (De) duct epithelial cell, (Ds) duct sheath cell, (En) endodermis, (Ep) epidermis, (Hy) hypodermis, (Gc) guard cell, (parenchymatic cell, (Ph) phloem, (Su) subsidiary cell, (Tp) transfusion parenchyma, (Tt) transfusion tracheid and, (Xy) xylem.


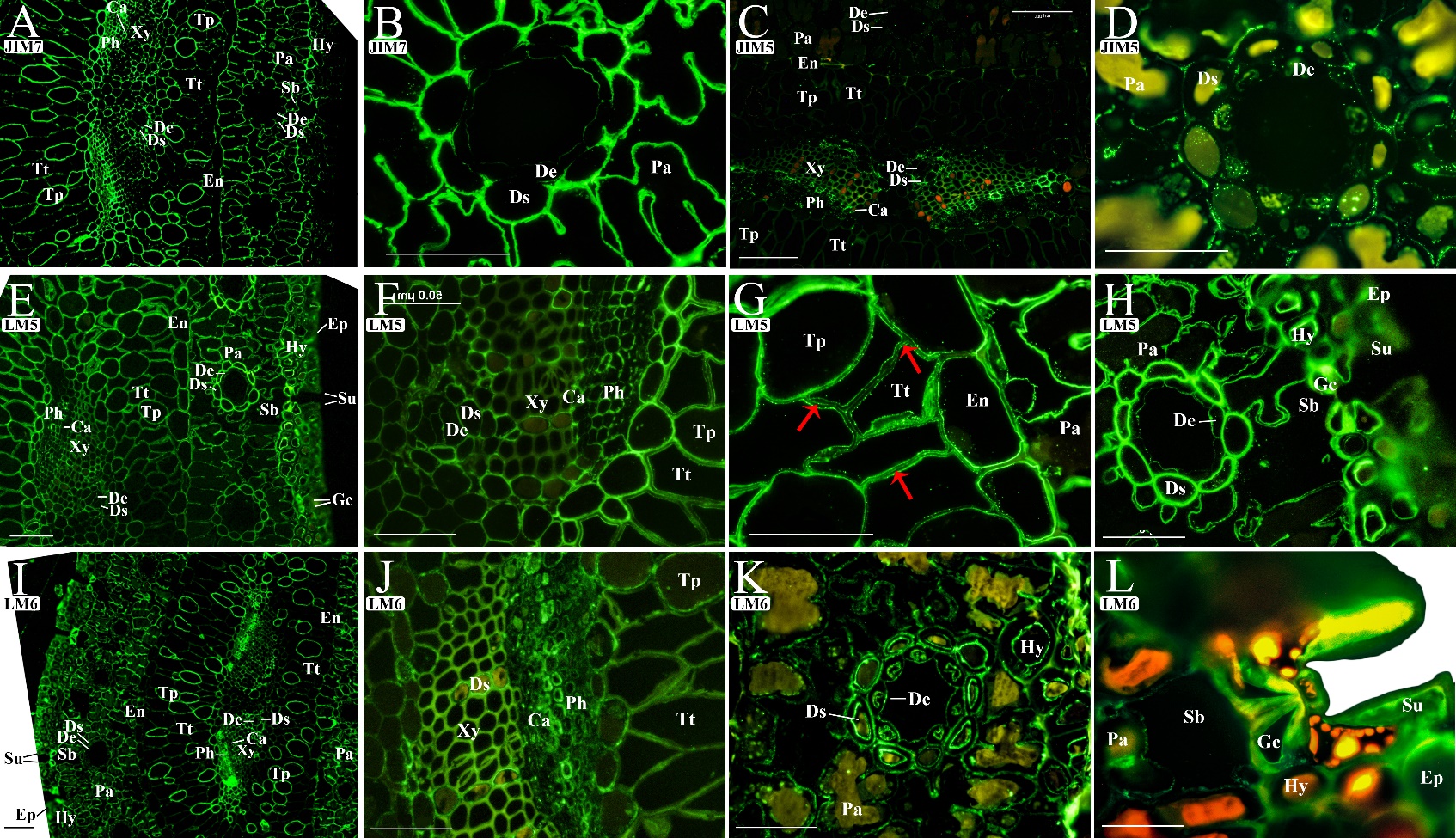


**Supplementary Figure 5.-** **Immunolocalization of pectin polysaccharides** in transverse sections of maritime pine (*Pinus pinaster*) needles using antibodies (green) that specifically labelled homogalacturonan with high – JIM7 (A general section, B mesophyll)—and low – JIM5 (C general section, D mesophyll)— degree of methyl esterification. and (1→4)-β-galactan – LM5 (E general section, F vascular bundle, G endodermis, H mesophyll and dermis)—and (1→5)-α-arabinan – LM6 (I general section, J vascular bundle, K mesophyll, L dermis)— side-chains of rhamnogalacturonan I. The red arrows indicate immunolabeling in middle lamella. Abbreviations: (Ca) cambium, (De) duct epithelial cell, (Ds) duct sheath cell, (En) endodermis, (Ep) epidermis, (Hy) hypodermis, (Gc) guard cell, (Pa) parenchymatic cell, (Ph) phloem, (Sb) substomatal chamber, (Su) subsidiary cell, (Tp) transfusion parenchyma, (Tt) transfusion tracheid and, (Xy) xylem. Scale bars of A, C, E, and I 100 µm; and for B, D, F, G, H, J, K, and L50 µm.

**
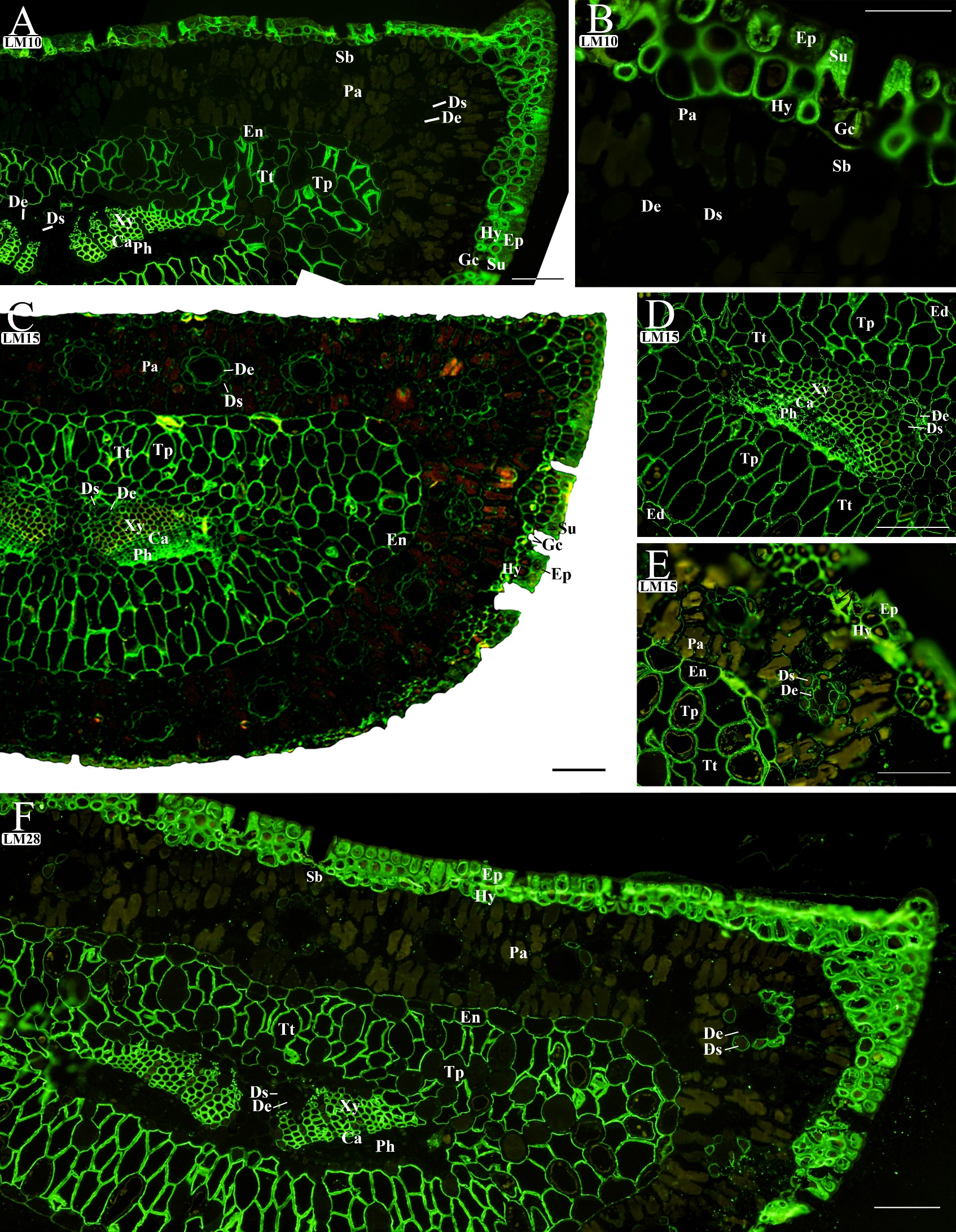
**

**Supplementary Figure 6.-** **Immunolocalization of hemicellulose polysaccharides** in transverse sections of maritime pine (*Pinus pinaster*) needles using the antibodies (green) that specifically labelled xylans – LM10— (A general section, B mesophyll and dermis), xyloglucan – LM15 (C general section, D central cylinder, E central cylinder, mesophyll and dermis)— and glucuronoxylan – LM28 (F general section)—. The autofluorescence shown in yellow and red is due to lignin, suberin or another cell wall compound. Abbreviations: (Ca) cambium, (De) duct epithelial cell, (Ds) duct sheath cell, (En) endodermis, (Ep) epidermis, (Hy) hypodermis, (Gc) guard cell, (Pa) parenchymatic cell, (Ph) phloem, (Sb) substomatal chamber, (Su) subsidiary cell, (Tp) transfusion parenchyma, (Tt) transfusion tracheid and, (Xy) xylem. All scale bars 100 µm.

**
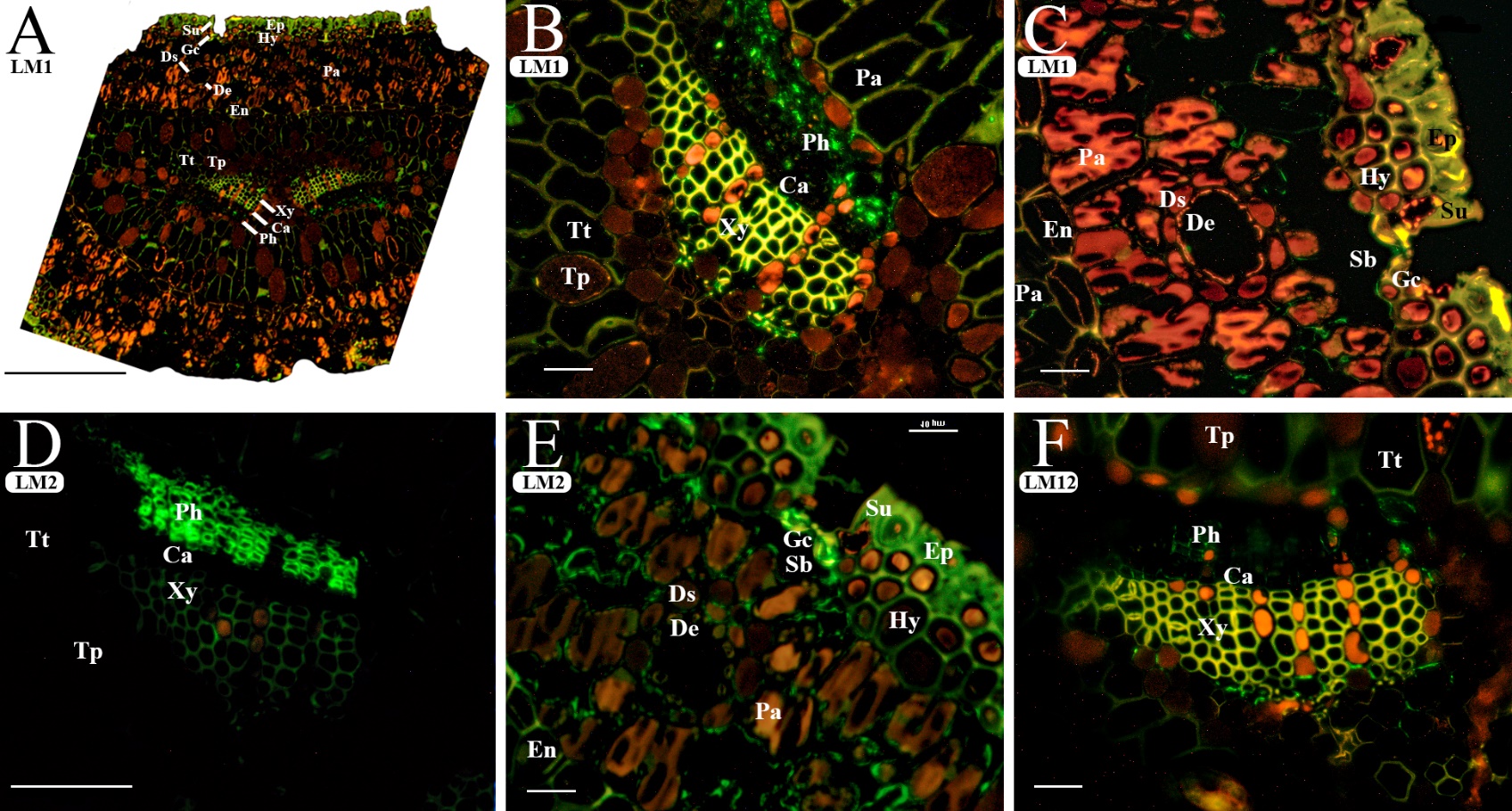
**

**Supplementary Figure 7.-** **Immunolocalization of cell wall proteins and ferulated polysaccharides** in transverse sections of maritime pine (*Pinus pinaster*) needles using the antibodies (green) that specifically label extension – LM1 (A general section, B vascular bundle, C mesophyll and dermis)—, arabinogalactan proteins –LM2 (D vascular bundle, E mesophyll and dermis)— and feruloylated polysaccharides –LM12 (F vascular bundle)—. The autofluorescence shown in yellow and red is due to lignin, suberin or another cell wall compound. Abbreviations: (Ca) cambium, (De) duct epithelial cell, (Ds) duct sheath cell, (En) endodermis, (Ep) epidermis, (Hy) hypodermis, (Pa) parenchymatic cell, (Ph) phloem, (Sb) substomatal chamber, (Su) subsidiary cell, (Tp) transfusion parenchyma, (Tt) transfusion tracheids and, (Xy) xylem. Scale bars of A and D 100 µm, D, C, E, and F 10 µm.


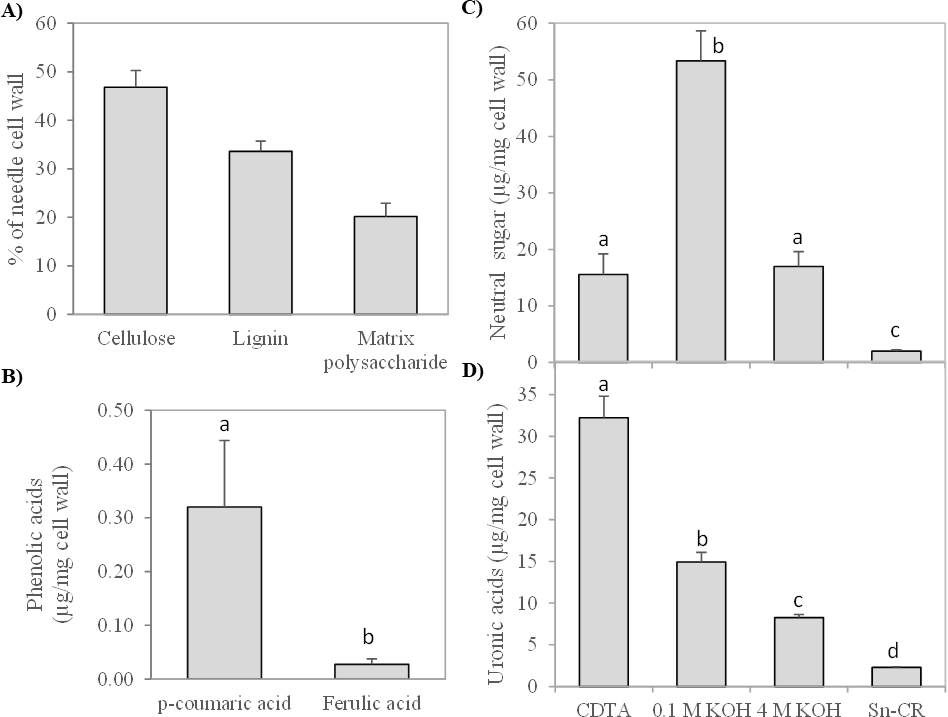


**Supplementary Figure 8.- Analysis of cell wall components of maritime pine (*Pinus pinaster*) needle**. (A) Cellulose, lignin and matrix polysaccharides. (B) Phenolic acids *p*-coumaric and ferulic. Content of (C) neutral sugars and (D) uronic acids in different cell wall fractions: CDTA, 0.1 M and 4 M KOH and Sn-CR. Data represent mean ± SE (n = 3 from 2 biological replicates). Letters indicate significant differences by (B) Student’s *t*-test (p<0.05) and (C, D) ANOVA (p<0.05). The fractionation yield was 43%.


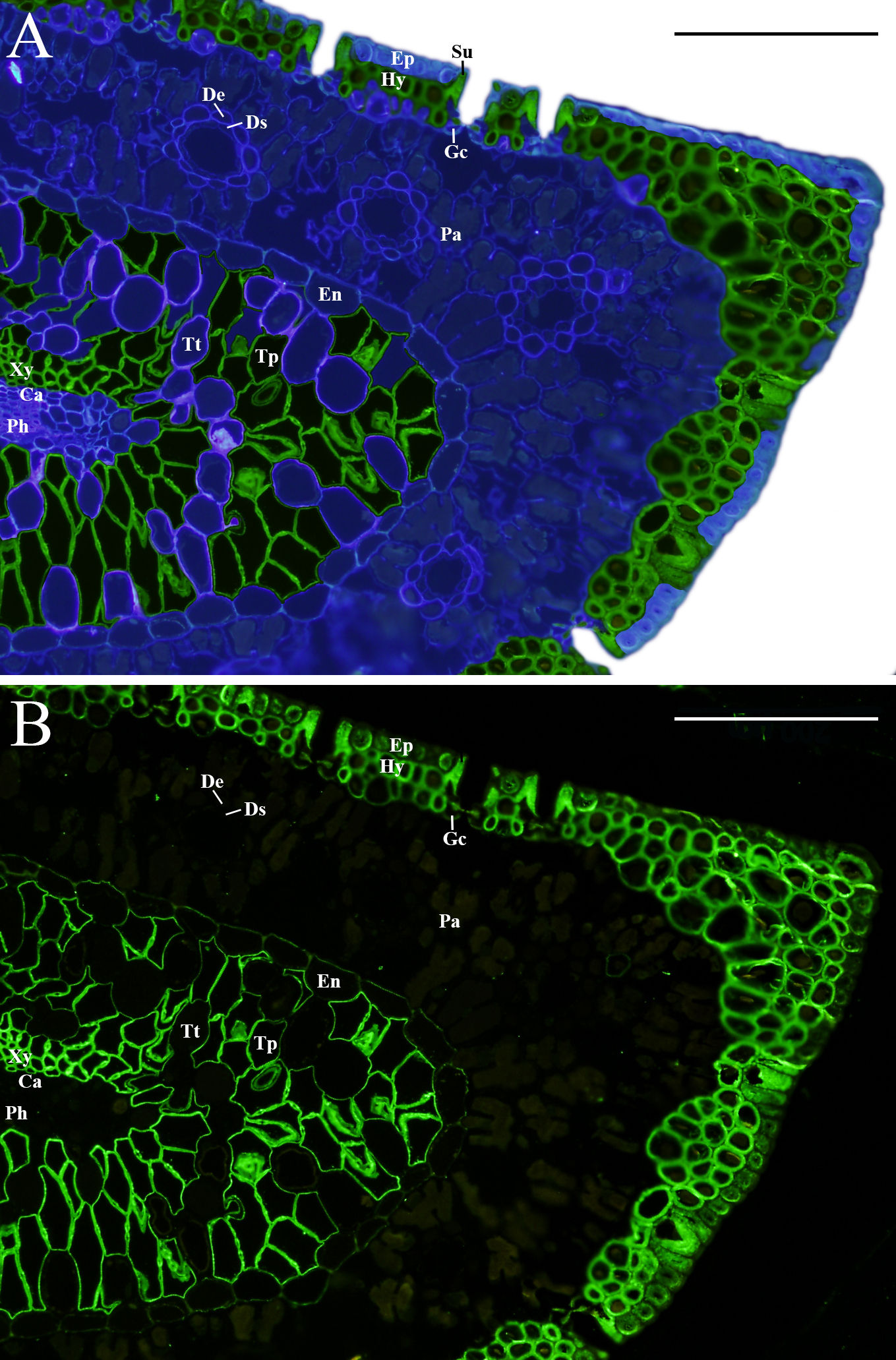


**Supplementary Figure 9.- Immunolocalization of xylans using LM11 antibody** (green) in transverse general section of maritime pine (*Pinus pinaster*) needles (A). The image has been edited with the same image taken for the calcofluor filter (blue) to better visualize the antibody binding (green) (B). Abbreviations: (Ca) cambium, (De) duct epithelial cell, (Ds) duct sheath cell, (En) endodermis, (Ep) epidermis, (Hy) hypodermis, (Gc) guard cell, (Pa) parenchymatic cell, (Ph) phloem, (Su) subsidiary cell, (Tp) transfusion parenchyma, (Tt) transfusion tracheid and, (Xy) xylem. Scale bar 200 µm. Note that the immunofluorescence pattern is similar to that found for the same as LM10 (see Fig. 4).
